# 11-deoxycortisol, other hormones and pituitary function characteristics in the diagnosis of adrenal insufficiency in the overnight oral metyrapone test

**DOI:** 10.1530/EC-26-0261

**Published:** 2026-08-10

**Authors:** Alexander Krawzow, Mirko Peitzsch, Manuela Bastian, Till Postrach, Lisa Johanna Harms, Elena Novikova, Graeme Eisenhofer, Holger S Willenberg

**Affiliations:** ^1^ Division of Endocrinology and Metabolism, Department of Medicine, Rostock University Medical Center, Rostock, Germany; ^2^ Institute of Clinical Chemistry and Laboratory Medicine, Faculty of Medicine and University Hospital Carl Gustav Carus, Technische Universität Dresden, Dresden, Germany; ^3^ Institute for Clinical Chemistry and Laboratory Medicine, Rostock University Medical Center, Rostock, Germany; ^4^ Department of Medicine III, University Hospital Carl Gustav Carus, Technische Universität Dresden, Dresden, Germany

**Keywords:** adrenal insufficiency, pituitary tumor, metyrapone, cortisol, steroid hormones

## Abstract

**Abstract:**

The oral metyrapone test is utilized to assess pituitary adrenal axis function. We asked whether 17-hydroxyprogesterone, androstenedione or corticotropin are similarly as useful as 11-deoxycortisol to detect adrenal insufficiency. Of the SHIP-PAGE study participants, 190 patients underwent an oral metyrapone test. The diagnosis of adrenal insufficiency included information from the clinical course, concentrations of adrenal steroids and ACTH before or after metyrapone as well as results of other pituitary function tests. Of the 190 patients, 67 had adrenal insufficiency and 123 had not. ROC analysis showed the highest sensitivity (83%), specificity (>90%) and area under the curve (0.9) for 11-deoxycortisol at a cutoff of 197 nmol/L. Surges in 11-deoxycortisol were related to those of 17-hydroxyprogesterone, androstenedione and corticotropin that displayed lower sensitivities, specificities and areas under the curve with cutoff values of 5.55 nmol/L, 8.11 nmol/L and 89.60 pg/mL, respectively. The presence of gonadal dysfunction had a high sensitivity of 61% and prolactin deficiency a high specificity (96%) for prediction of adrenal insufficiency as had a combined dysfunction of the gonadal, growth hormone and thyroid axes (97%). In conclusion, 11-deoxycortisol is an already established parameter with a good performance in the oral metyrapone test, which was not outperformed by 17-hydroxyprogesterone, androstenedione or corticotropin. However, there may be a substantial bias to this result. Therefore, determination of pituitary–end-organ axes functions is likewise helpful to provide probability information in the assessment of adrenal insufficiency, which remains a diagnosis based on clinical information and endocrine function tests.

**Significance statement:**

The oral metyrapone test is very useful for the characterization of adrenal function. If available, analysis of 11-deoxycortisol is better than measurements of other adrenal steroid hormones or corticotropin. However, there may be a bias from previous studies on the metyrapone test. Therefore, analysis of corticotropin, androstenedione and 17-hydroxyprogesterone concentrations at baseline and the day after metyrapone administration is also very helpful for the evaluation of adrenal function when the results from other pituitary function tests are included in the diagnostic process.

## Introduction

Metyrapone inhibits the enzyme 11β-hydroxylase (CYP11B1), which is found in the adrenal cortex. Inhibition of 11β-hydroxylase activity prevents the conversion of 11-deoxycortisol (11S) to cortisol (F) and the conversion of 11-deoxycorticosterone (DOC) to corticosterone in the adrenal cortex. Metyrapone was used to characterize this effect by Liddle *et al.* (1) and later evolved into a test for the diagnosis of adrenocortical insufficiency (2, 3). After oral metyrapone administration, plasma cortisol concentrations drop due to a diminished conversion of 11S to cortisol, which interrupts the negative feedback by cortisol onto hypothalamic and anterior pituitary cells. As a consequence, generation of corticotropin-releasing hormone (CRH) and corticotropin (ACTH) increases in normal individuals. The surge in ACTH leads to stimulation of the steroidogenesis in the adrenal cortex and an increase in 11S (1). Plasma spikes of 11S after metyrapone can be used for diagnosing the function of the hypothalamic–pituitary–adrenal (HPA) axis and to characterize adrenal insufficiency (AI). For oral metyrapone test (OMT), an 11S increase over 200 nmol/L was defined as an indicator for normal HPA axis and adrenocortical function (4). The cutoff of >200 nmol/L for 11S is often used in the context of OMT (5, 6). An increase in the adrenocortical hormone 17α-hydroxyprogesterone (17OHP) after metyrapone administration could already be shown in some studies (7, 8). Furthermore, Auzéby *et al.* observed an increase in the adrenocortical hormone androstenedione (Δ4A’dione) as a consequence of 11β-hydroxylase blockade by MET during their *in vitro* study (9). In people with normal pituitary adrenal function, different ACTH responses were observed in OMT, such as 200 pg/mL (10) or 150 pg/mL (33 pmol/L) (11). Since immunoassays to 17OHP, Δ4A’dione and ACTH are more widely distributed than assays for measuring 11S, we asked whether the determination of 17OHP, Δ4A’dione or ACTH in OMT can be similarly as useful as 11S to biochemically detect AI. We also collected data on pituitary axis function to address the question to what extent clinical information on hypopituitarism can also be indicative of adrenal insufficiency in comparison with hormone function tests.

## Patients and methods

### Patients

As part of the SHIP-PAGE study program (steroid hormones in patients with pituitary adrenal or gonadal endocrinopathies), which was approved by the local ethical committee, patients who are studied for adrenal insufficiency undergo endocrine function testing, including the OMT during the diagnostic process. Starting in 2015, patients could be constitutively included in the data analysis (12, 13). Patients with primary adrenal insufficiency were excluded from this study. Consent was obtained from each patient or subject after full explanation of the purpose and nature of all procedures used. The vast majority of patients have been evaluated clinically following an inpatient and outpatient protocol.

### Biochemical methods

Serum cortisol and plasma ACTH were measured on the Cobas platform (Roche Diagnostics GmbH, Germany), employing the Elecsys^®^ Cortisol II and Elecsys® ACTH assays. 11S, 17OHP and Δ4A’dione were measured by tandem mass spectrometry using calibrators from a commercial kit provided by Chromsystems (Chromsystems Instruments & Chemicals GmbH, Germany).

### Study protocol and statistical analysis

As part of OMT, patients received 2 g metyrapone orally at 23:00 h and blood was drawn at 08:00 h the next morning for determination of serum cortisol, 11S, 17OHP and Δ4A’dione and plasma ACTH (F-OMT, 11S-OMT, 17OHP-OMT, Δ4A’dione-OMT and ACTH-OMT). In most patients, basal hormone concentrations were determined in the blood on the morning of the test day as part of the OMT (F-basal, 11S-basal, 17OHP-basal, Δ4A’dione-basal and ACTH-basal). Patients who were under hydrocortisone replacement therapy paused it on the test day and after 16:00 h the evening before. After asservation of blood samples, patients took 10 mg hydrocortisone in the morning and 5 mg at noon after the metyrapone test.

In some of the patients, further endocrine tests, such as ACTH stimulation test (ACTH-test), insulin hypoglycemia test (IHT) or a corticotropin-releasing hormone challenge (CRH-test), had been carried out. An 11S increase in OMT to >200 nmol/L was considered as an indicator of a formal sufficient adrenocortical response. However, for determining whether a patient had AI or not, we took all the information from outpatient and inpatient care available into consideration, including clinical aspects, such as extent of deficiencies of other hormonal axes of the anterior pituitary, clinical course, problems of serum sodium, necessity of adrenal steroid hormone replacement, ACTH concentrations at baseline and after OMT and, in some cases, results of other endocrine function tests. In addition, clinical data and results of other pituitary function tests have been collected on the basis of the Endocrine Society Clinical Practice Guideline to judge on the capacity of other pituitary axes whereby tumor sizes and response to therapy have also been taken into account (14).

Gonadal insufficiency was defined as a low testosterone concentration with inappropriately low or normal LH and/or FSH levels, in line with the Endocrine Society Clinical Practice Guideline by Fleseriu *et al.* (14).

In addition to the biochemical definition, we defined hypogonadotropic hypogonadism according to Campana *et al.*, taking into account clinical features, including menstrual disturbances, infertility and reduced libido in women, as well as erectile dysfunction, reduced libido and impaired spermatogenesis in men (15).

Pituitary function was assessed before the start of any pituitary-directed therapy, including dopamine agonists and somatostatin receptor ligands. Prolactin levels were interpreted in a broader clinical context, considering pituitary disease, other pituitary hormone axes, imaging results and clinical history. Medication effects on prolactin were excluded based on the patients’ medical records.

Furthermore, several studies have attempted to define hypoprolactinemia based on biochemical prolactin concentrations, using a cutoff of approximately 5 ng/mL, which we also adopted in our study (16, 17).

Growth hormone (GH) deficiency was diagnosed based on serum insulin-like growth factor 1 (IGF-1) concentrations, GH-stimulation testing, including the GH-releasing hormone–arginine test, and the presence of additional pituitary hormone deficiencies, according to the Endocrine Society Clinical Practice Guideline (18).

We created receiver operator characteristics for F-basal, 11S-basal, 17OHP-basal, Δ4A’dione-basal and ACTH-basal and for 11S-OMT, ACTH-OMT, 17OHP-OMT, Δ4A’dione-OMT and ACTH-OMT with SPSS Statistics (IBM Statistical Package for Social Science, USA). Using the Youden indices, we were able to determine cutoffs with the best combination of sensitivity (SEN) and specificity (SPEC) in each case. For the comparison of areas under the ROC curves (AUCs), we used MedCalc statistical software (MedCalc Software Ltd, Belgium) and the method of DeLong (19). Boxplots were generated using Origin software (OriginLab corporation, USA). To evaluate whether there was a statistically significant difference in 11S-OMT, 17OHP-OMT, Δ4A’dione-OMT and ACTH-OMT between the group of patients without adrenal insufficiency and those with adrenal insufficiency, the Mann–Whitney U test was performed. The corrected significance level after Bonferroni for the Mann–Whitney U test was *P* = 0.0125.

The data for the hormones in OMT were non-normally distributed. Therefore, to analyze associations, correlations after Spearman have been determined in SPSS. Using the information from the clinical course along with basal blood concentrations of follicle-stimulating hormone (FSH), luteinizing hormone (LH), testosterone, estradiol, prolactin, GH, IGF-1, thyroid-stimulating hormone (TSH), thyroxine (T4) and triiodothyronine (T3) and endocrine function tests, we assessed gonadotroph, prolactin, growth hormone and thyrotropin functions. Employing the information of hypothalamic–end-organ–axis functions, we could determine the sensitivities (SEN), specificities (SPEC), positive predictive values (PPVs) and negative predictive values (NPVs) for the prediction of AI.

To compare the characteristics of patients with and without adrenal insufficiency for statistically significant differences, we used Fisher’s exact test.

## Results

### Patients

Between 2015 and 2025, 190 patients were consecutively investigated for adrenal insufficiency at our clinic using the OMT. Of them, 58% were female. The majority was diagnosed with pituitary adenoma (PIT-NET) (Table 1). The mean value of body mass index for all patients was 27.7 kg/m^2^. In our cohort, patients with AI demonstrated a higher mean body weight (Table 1).

**Table 1:** Main patient characteristics.

	All patients	Patients without AI	Patients with AI	*P*-values
Patients, *n*	190	123	67	<0.001
Age, median (IQR)	59 (41–69)	60 (47–71)	55 (39–68)	n.s.
Females, *n*	111 (58%)	81 (66%)	30 (45%)	<0.01
Body mass index (kg/m^2^)	27.7 ± 5.7	26.8 ± 5.4	29.1 ± 6.0	n.s.
Systolic blood pressure (mmHg)	124.1 ± 5.7	122.3 ± 17.2	126.9 ± 21.5	n.s.
Diastolic blood pressure (mmHg)	73.9 ± 11.4	72.4 ± 10.2	76.3 ± 12.9	n.s.
CPA	6	3	3	–
Craniopharyngioma	3	3	0	–
Glioma or meningioma	4	3	1	–
Glucose metabolism disorders	6	5	1	–
Hypophysitis	4	2	2	–
Idiopathic hypopituitarism	5	3	2	–
Langerhans’ cell histiocytosis	2	1	1	–
PAS 1 and PAS 2	2	2	0	–
PIT-NET after surgery	62	33	29	<0.05
PIT-NET non-operated	29	20	9	n.s.
Pituitary cyst after surgery	4	4	0	–
Pituitary cyst non-operated	5	5	0	–
Tertiary adrenal insufficiency	18	11	7	n.s.
Others	40	28	12	n.s.
No additional deficiency	108 (57%)	86 (70%)	22 (33%)	<0.001
One additional pituitary deficiency	27 (14%)	21 (17%)	6 (9%)	n.s.
Two additional pituitary deficiencies	30 (16%)	12 (10%)	18 (27%)	<0.01
Three additional pituitary deficiencies	20 (11%)	4 (3%)	16 (24%)	<0.001
Four additional pituitary deficiencies	5 (2%)	0 (0%)	5 (7%)	<0.01

In all patients, no side effects were reported following the OMT.

Almost two-thirds of all patients have been judged to have intact hypothalamic–pituitary–end organ function. Combined dysfunction of pituitary–gonadal, prolactin, GH–IGF-1 and pituitary–thyroid functions is predictive of AI with a specificity of 100% (Table 2). Gonadal dysfunction exhibited the highest sensitivity of 61%, while prolactin deficiency showed the highest specificity of 96% for a single predictor. Hypofunction of at least one hypothalamic–pituitary axis yielded a sensitivity of 67% (Table 2).

**Table 2:** Hypothalamic–pituitary–end-organ axis failures, sensitivities and specificities.

	SEN (%)	SPEC (%)	PPV (%)	NPV (%)
Gonadal axis	61	77	61	77
Lactotroph axis	13	96	67	66
Somatotropic axis	56	82	65	77
Thyroid axis	41	93	76	73
Failure of at least one axis	67	70	55	80
Failure of one axis	9	83	19	63
Failure of two axes	27	90	60	70
Failure of three axes	24	97	75	70
Failure of four axes	7	100	100	66

### Results from the biochemical studies

**Figure 1:**
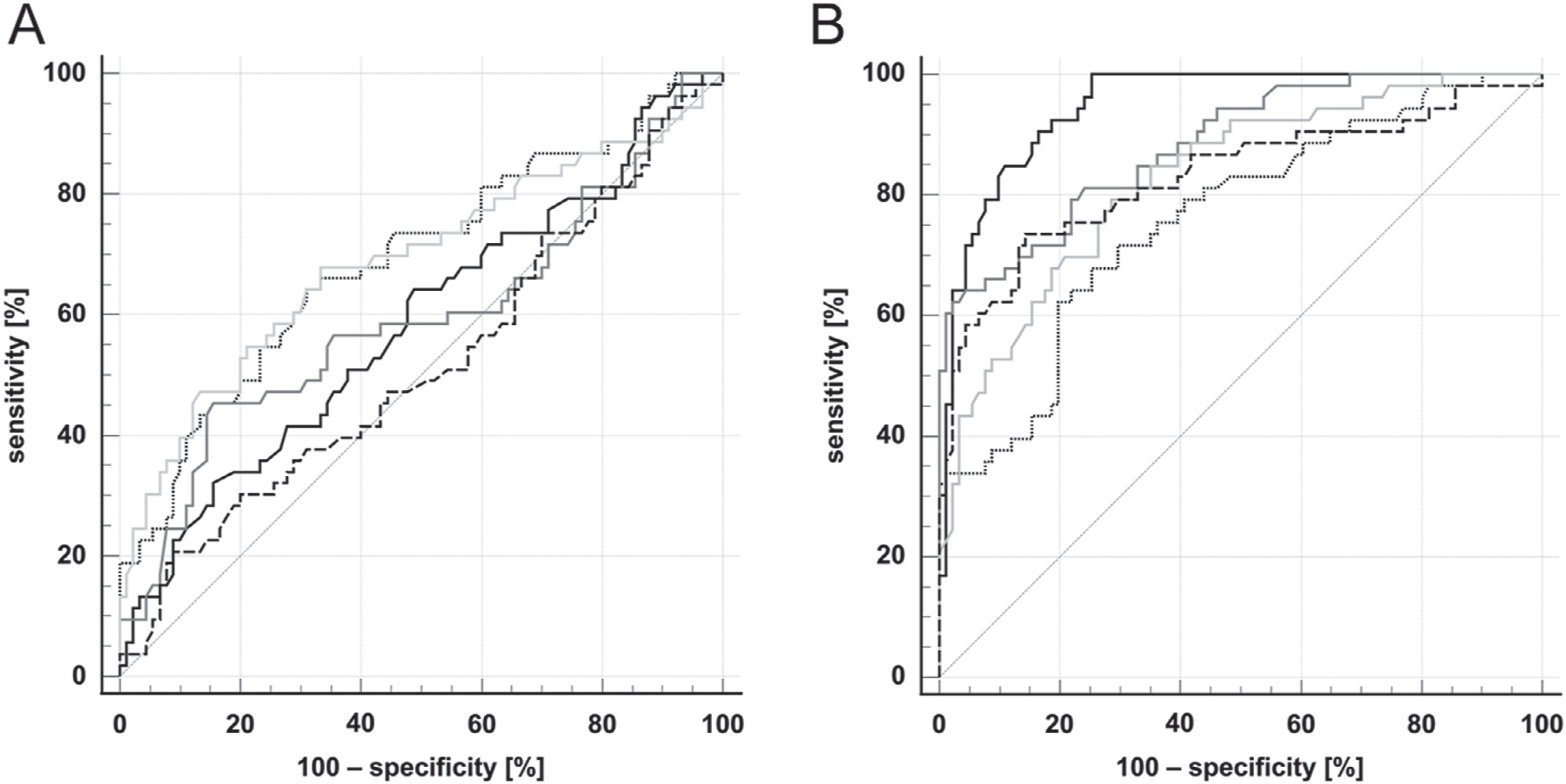
(A) Receiver operating characteristic (ROC) curves of basal cortisol, 11-deoxycortisol, 17α-hydroxyprogesterone, androstenedione and corticotropin (F-basal, 11S-basal, 17OHP-basal, Δ4A’dione-basal, ACTH-basal, respectively). Sensitivity is plotted on the y-axis, and 1 − specificity is plotted on the x-axis. F-basal shows the highest area under curve (AUC) of 0.712 followed by Δ4A’dione-basal with an AUC of 0.694. ACTH-basal shows the lowest AUC with a value of 0.508. The AUC of Δ4A’dione-basal is significantly different than that of ACTH-basal (*P* < 0.05), but not significantly different than the AUC from F-basal according to the comparison of AUCs by the method of DeLong. The values for the corresponding AUC for these ROC curves are presented in Table 2. Legend to the ROC curves: black continuous line – 11S-basal, black dotted line – F-basal, black dashed line – ACTH-basal, dark gray line – 17OHP-basal, and light gray line –Δ4A’dione-basal. (B) Receiver operating characteristic (ROC) curves of cortisol, 11-deoxycortisol, 17α-hydroxyprogesterone, androstenedione and corticotropin after metyrapone administration (F-OMT, 11S-OMT, 17OHP-OMT, Δ4A’dione-OMT and ACTH-OMT, respectively). Sensitivity is plotted on the y-axis, and 1 − specificity is plotted on the x-axis. The curve of 11S-OMT shows the largest area under the curve (AUC) with a value of 0.907, followed by the AUC of Δ4A’dione-OMT with a value of 0.825. The AUC of 11S-OMT is significantly different than that of 17OHP-OMT (*P* < 0.05), Δ4A’dione-OMT (*P* < 0.01) and ACTH-OMT (*P* < 0.01) according to the method of DeLong. The values for the corresponding AUC for these ROC curves are presented in Table 2. Legend to the ROC curves: black continuous line – 11S-OMT, black dotted line – F-OMT, black dashed line – ACTH-OMT, dark gray line – 17OHP-OMT, and light gray line –Δ4A’dione-OMT.

**Figure 2:**
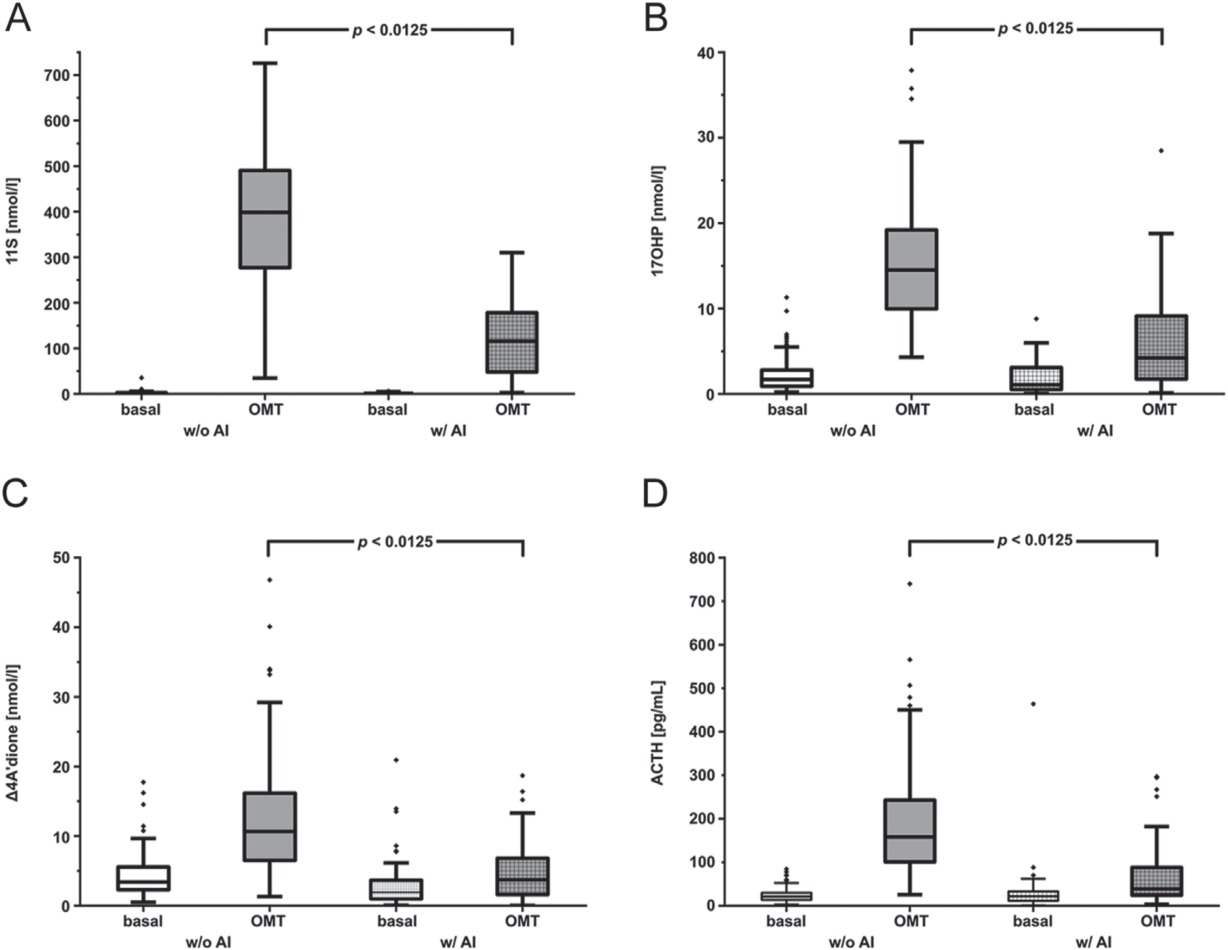
(A, B, C, D) Boxplots for 11-deoxycortisol (11S, A), 17α-hydroxyprogesterone (17OHP, B), androstenedione (Δ4A’dione, C) and ACTH (corticotropin, D) for patients without adrenal insufficiency (without AI) and with adrenal insufficiency (with AI) at baseline and after OMT. The medians of 11S, 17OHP, Δ4A’dione and ACTH after OMT are higher for patients without AI than for patients with AI. Box colors for patients without AI are white at baseline and light gray after OMT. Box colors for patients with AI show a grid pattern on white at baseline and a grid pattern on light gray after OMT.

ROC analysis showed that basal determinations of adrenal steroid hormones and basal ACTH determinations had low sensitivities (SEN) and specificities (SPEC) as also shown by the Youden indices (Table 3; Fig. 1, panel A). The best performance was seen with the determination of 11S-OMT at a cutoff of 197 nmol/L that rendered comparable a high SEN (83%) and SPEC (>90%) and an area under the curve (AUC) of 0.907 (Table 3; Fig. 1, panel B). The AUC of 11S-OMT was found to be significantly different in comparison with that of 17OHP-OMT (0.734) (*P* < 0.05), Δ4A’dione-OMT (0.825) (*P* < 0.01) and ACTH-OMT (0.835) (*P* < 0.05) (Table 3; Fig. 1, panel B). The AUC of Δ4A’dione-basal (0.694) differed significantly from that of ACTH-basal (0.508) (*P* < 0.05) but not from F-basal (0.712) (Table 3; Fig. 1, panel A). In contrast to 11S-OMT, 17OHP-OMT and Δ4A’dione-OMT showed a lower SEN and SPEC. Calculation of SEN for 17OHP-OMT yielded 62% and a SPEC of 96%. Compared with 17OHP-OMT, Δ4A’dione-OMT showed a slightly better SEN (85%) and worse SPEC (64%, Table 3). Δ4A’dione-OMT showed a higher AUC (0.825) than 17OHP-OMT (0.734) (Fig. 1, panel B). ACTH-OMT had a SEN of 76% and a SPEC of 82% with an AUC of 0.835 at a cutoff of 89.6 pg/mL (Table 3; Fig. 1, panel B). The highest Youden index exhibited 11S-OMT determinations (0.77), followed by ACTH-OMT (0.58), 17OHP-OMT (0.58) and Δ4A’dione-OMT (0.49) (Table 3). We found statistically significant differences for 11S-OMT, 17OHP-OMT, Δ4A’dione-OMT and ACTH-OMT between patients with adrenal insufficiency and those without AI (*P* < 0.0125) (Fig. 2, panels A, B, C, D).

**Table 3:** Cutoff values, sensitivities and specificities as determined by ROC curve analysis.

	Cutoff	Concentration	SEN (%)	SPEC (%)	Youden	PPV (%)	NPV (%)	AUC
F-basal (nmol/L)	Lower limit	N/A	N/A	N/A	N/A	N/A	N/A	**0.712**
	Optimal	232.50	53	80	0.33	59	76	
	Upper limit	N/A	N/A	N/A	N/A	N/A	N/A	
F-OMT (nmol/L)	Lower limit	59.40	62	82	0.44	64	80	**0.767**
	Optimal	67.05	68	77	0.45	61	81	
	Upper limit	71.40	71	73	0.44	58	82	
11S-basal (nmol/L)	Lower limit	N/A	N/A	N/A	N/A	N/A	N/A	**0.604**
	Optimal	1.28	58	59	0.18	43	73	
	Upper limit	N/A	N/A	N/A	N/A	N/A	N/A	
11S-OMT (nmol/L)	Lower limit	175.00	74	96	0.70	91	87	**0.907**
	Optimal	196.68	83	93	0.77	87	91	
	Upper limit	252.55	94	82	0.76	74	96	
17OHP-basal (nmol/L)	Lower limit	N/A	N/A	N/A	N/A	N/A	N/A	**0.581**
	Optimal	0.74	44	86	0.29	63	73	
	Upper limit	N/A	N/A	N/A	N/A	N/A	N/A	
17OHP-OMT (nmol/L)	Lower limit	4.89	59	99	0.58	59	81	**0.734**
	Optimal	5.55	62	96	0.58	62	82	
	Upper limit	7.34	67	88	0.56	61	83	
Δ4A’dione-basal (nmol/L)	Lower limit	N/A	N/A	N/A	N/A	N/A	N/A	**0.694**
	Optimal	2.59	68	68	0.36	54	79	
	Upper limit	N/A	N/A	N/A	N/A	N/A	N/A	
Δ4A’dione-OMT (nmol/L)	Lower limit	7.01	78	71	0.49	60	85	**0.825**
	Optimal	8.11	85	64	0.49	57	88	
	Upper limit	9.33	87	59	0.46	55	89	
ACTH-basal (pg/mL)	Lower limit	N/A	N/A	N/A	N/A	N/A	N/A	**0.508**
	Optimal	10.75	24	89	0.13	55	68	
	Upper limit	N/A	N/A	N/A	N/A	N/A	N/A	
ACTH-OMT (pg/mL)	Lower limit	72.40	72	84	0.56	72	84	**0.835**
	Optimal	89.60	76	82	0.58	70	86	
	Upper limit	96.90	79	77	0.56	65	86	

### Correlations of hormonal parameters of pituitary–adrenal–function

Statistically significant and associations were observed between all measured steroid hormones and between them and ACTH, except for F-basal and 11S-basal, ACTH-basal and 11S-basal as well as ACTH-basal and 17OHP-basal (Table 4). The highest correlations were observed between 11S-OMT and 17OHP-OMT (Spearman-*r* = 0.660, *P* < 0.05), 11S-OMT and Δ4A’dione-OMT (Spearman-*r* = 0.624, *P* < 0.05) as well as 11S-OMT and ACTH-OMT (Spearman-*r* = 0.601, *P* < 0.01) (Table 4).

**Table 4:** Correlations between hormonal parameters of pituitary–adrenal function.

		F-OMT	11S-basal	11S-OMT	17OHP-basal	17OHP-OMT	Δ4A’dione-basal	Δ4A’dione-OMT	ACTH-basal	ACTH-OMT
F-basal	Spearman-*r*	0.386	0.451	0.339	0.306	0.319	0.336	0.260	0.311	0.286
	*P*-value	<0.001	<0.001	<0.001	<0.001	<0.001	<0.001	0.001	<0.001	<0.001
F-OMT	Spearman-*r*		0.011	0.381	0.221	0.460	0.327	0.497	0.161	0.349
	*P*-value		0.892	<0.001	0.007	<0.001	<0.001	<0.001	0.029	<0.001
11S-basal	Spearman-*r*			0.334	0.393	0.313	0.352	0.056	0.156	0.121
	*P*-value			<0.001	<0.001	<0.001	<0.001	<0.001	0.042	<0.001
11S-OMT	Spearman-*r*				0.212	0.660	0.336	0.624	0.071	0.601
	*P*-value				0.009	<0.001	<0.001	<0.001	0.340	<0.001
17OHP-basal	Spearman-*r*					0.492	0.595	0.245	0.244	0.187
	*P*-value					<0.001	<0.001	0.003	0.003	0.022
17OHP-OMT	Spearman-*r*						0.488	0.431	0.067	0.598
	*P*-value						<0.001	<0.001	0.408	<0.001
ΔA4’dione-basal	Spearman-*r*							0.460	0.326	0.350
	*P*-value							<0.001	<0.001	<0.001
ΔA4’dione-OMT	Spearman-*r*								0.218	0.463
	*P*-value								0.008	<0.001
ACTH-basal	Spearman-*r*									0.291
	*P*-value									<0.001

## Discussion

Diagnosis of AI is thought to occur in 1.5–3 per 10.000 subjects (20), a majority of which have pituitary disease and show excess mortality (21, 22). Therefore, correct diagnosis should be made because even mild cases may experience severe adrenal failure during stress (23). For an accurate diagnosis of AI, endocrine function tests are required along with information from the case history and clinical course (24). Since the OMT relies on measurements of 11S and assays for 11S have not been ubiquitary available, we evaluated whether 17OHP, Δ4A’dione and ACTH were useful parameters in addition to 11S in the OMT for biochemical diagnosis of AI.

ROC analysis rendered a cutoff of 197 nmol/L for 11S-OMT, which is close to the cutoff of 200 nmol/L reported in previous studies (5, 6, 11). However, this expresses a selection bias rather than a proof of previous study results. Along this line, 11S-OMT revealed the best performance in OMT as compared to ACTH-OMT, Δ4A’dione-OMT and 17OHP-OMT with respect to SEN, SPEC, PPV, NPV, Youden’s index and AUC. The outcome of 11S-OMT was not much changed by inclusion of clinical data or the information from other tests. However, direct comparisons with other tests are needed to come to better conclusions.

Notably, both Δ4A’dione-OMT and 17OHP-OMT showed significant correlations with 11S-OMT although Δ4A’dione-OMT and 17OHP-OMT yielded much lower Youden’s indices. Thus, we conclude that, currently, 17OHP-OMT and Δ4A’dione are less suitable parameters than 11S-OMT in OMT. In a study from 1985, Laurberg *et al.* had also attempted to evaluate 17OHP-OMT as a possible parameter in OMT, but noted that their patient group was not large enough and that a larger patient group was needed for a final evaluation. Concordant with our result, they assumed that 17OHP-OMT was not a useful parameter in OMT (8). Seoudy *et al.* showed in their study that patients with an 11S increase <200 nmol/L (70 μg/L) had significantly lower surges in Δ4A’dione and 17OHP after OMT as compared to the patient group with an increase in 11S ≥ 200 nmol/L (25).

Since ACTH had the second highest AUC after 11S-OMT in our analyses, we infer that ACTH appears helpful to some extent. In our ROC analysis, ACTH-OMT showed a better performance than Δ4A’dione-OMT and 17OHP-OMT in terms of sensitivity with the same Youden index as 17OHP-OMT. On the other hand, the overall performance of ACTH-OMT was clearly inferior to that of 11S-OMT and ACTH-OMT is a rather mediocre parameter in the OMT. Other studies lend support to ACTH-OMT as a parameter in addition to 11S-OMT and proposed different cutoffs for ACTH-OMT, such as 200 pg/mL (10) or 150 pg/mL (33 pmol/L) (11), which seemed rather high as compared to our analysis. In a more recent study, a cutoff value of 78.6 pg/mL (17.3 pmol/L) for ACTH-OMT was close to our analysis of 89.6 pg/mL (19.7 pmol/L) (26), while in the recent study by Noe *et al.*, the plasma ACTH concentrations of patients had been measured every sixty minutes for four hours after oral metyrapone administration. They found an ACTH-OMT cutoff of 86.7 pg/mL at 60 min or at 120 min, which is very close to our ACTH-OMT cutoff, and observed that measurements afterward had no additional benefit (27). In a study from 2022, a cutoff for ACTH-OMT of 147 pg/mL has been determined, with a SEN of 73.2% (28). In a similar study, a cutoff for ACTH in the OMT was found to be lower with 67.4 pg/mL, with a SEN of 83% and a SPEC of 80% (25). Thus, it can be seen that although the cutoff values for ACTH in some studies are similar, overall, they do differ in terms of cutoff and also SEN and SPEC, making it difficult to establish ACTH after OMT as a reliable biomarker for AI. It should be noted that the assays used for ACTH determination differed between the various studies, which may have contributed to the differences in the reported results. Several studies have highlighted that ACTH concentrations may vary depending on the assay used, as commercially available ACTH immunoassays differ in analytical performance and accuracy (29, 30). A related problem was described in a meta-analysis that compared cutoffs from different studies for basal cortisol concentrations and cortisol concentrations after ACTH challenge, showing that the range of these cutoffs is relatively wide (31). Thus, the lack of standardization in biochemical measurements of hormonal parameters may account for the differing cutoff values reported across studies.

A difference between our study and some others that addressed the determination of cutoffs for ACTH in OMT is that our ROC curves differed from their ROC curves in terms of what was used as a reference for judging whether patients had AI or not. For example, Berneis *et al.* presented different ROC curves for 11S and the sum of 11S and cortisol in which the assessment about a presence of AI was chosen by an ACTH response in the OMT, for example >150 pg/mL and >200 pg/mL (32). Others used the IHT as a reference test for their ROC curve to classify their patients with AI and without AI (26). For our assessment whether AI was present in the patients in our study or not, we considered all available information, as described earlier. Our assessment included clinical aspects, clinical course, glucocorticoid replacement need and ACTH concentrations at baseline and after OMT, and in many cases, also results of other endocrine function tests. This assessment was a requirement for creating our ROC curves and, therefore, not a mere test analysis compared to a golden standard.

In terms of performance of OMT, it is to be mentioned that all patients received 2 g metyrapone orally. Since no weight adjustments have been done, patients with AI happened to ingest a comparatively lower weight-adjusted metyrapone dose. However, as compared to patients with normal adrenocortical function, patients with AI had lower basal and metyrapone-inhibited cortisol concentrations. This indirectly shows that a dose of 2 g metyrapone is enough to sufficiently inhibit 11β-hydroxylase activity. Similarly, in the dexamethasone suppression test (DST), a dexamethasone dose of 1 mg is given regardless of body weight (33, 34). Several studies have shown that dose adaptation of dexamethasone to body weight had no relevant advantages (35, 36), and this may also apply to the OMT.

Cutoffs for all basal adrenal steroid hormones and for basal ACTH showed low SEN, SPEC, Youden’s indices and AUCs. Surprisingly, Δ4A’dione-basal exhibited the best Youden index compared to all basal hormones. Moreover, the SEN of Δ4A’dione-basal (68%) was higher than the SEN of F-basal (53%) and the AUCs from Δ4A’dione-basal and F-basal did not differ significantly. This is quite unexpected, since in many studies, F-basal is actually proposed as the first assessment whether the patient has AI or not. Lower cutoffs for morning basal cortisol are, for example, 98 nmol/L (37) or 110 nmol/L (4 μg/dl) (38). However, the cut-off of 232.50 nmol/L we determined showed only a low SEN of 53% and a mediocre SPEC of 80%.

Consideration of the number of failures of the hypothalamic–pituitary–end-organ axes, which include the gonadal, lactotroph, growth hormone, and thyroid axes, may be helpful in evaluating an AI. For example, in our study, failure of two of these hormonal axes showed a SPEC of 90% and failure of all four axes resulted in a maximal SPEC. Similarly, failure of the lactotroph axis showed a very high SPEC. In fact, the presence of other pituitary hormone deficiencies and pituitary surgery turned out to be significant predictors or risk factors to develop HPA axis insufficiency (39, 40).

It is of importance that a bias of the study results is introduced toward the definition and methods used and the present findings are to be interpreted against the background of metyrapone testing. Therefore, the diagnosis of adrenal insufficiency should extend beyond biochemical parameters alone in the future and include clinical features, such as disturbances in salt balance, low blood pressure and features in a medical history suggestive of adrenal insufficiency. Such an integrative approach is already applied in the diagnosis of adrenal crisis and may improve the assessment of the likelihood of adrenal insufficiency in patients undergoing the oral metyrapone test (41).

In summary, our results support the clinical practice that AI remains a diagnosis that is based very much on clinical information in combination with results of different endocrine function tests whereby 11S-OMT shows a satisfactory outcome in the characterization of patients despite a historically arisen bias and a protocol that includes inpatient care.

## Declaration of interest

The authors declare that there is no conflict of interest that could be perceived as prejudicing the impartiality of the work reported.

## Funding

AK received funding for this study through the FORUN program 2025 of the Rostock University Medical Centerhttps://www.med.uni-rostock.de/forschung-lehre/forschung/forschungsfoerderung (#889118).

## Ethical statement

This study was approved by Local Ethical Committee of the Medical Faculty of the Rostock University Medical Center (#A2016-0088).

## References

[1] Liddle GW, Island D, Lance EM, et al. Alterations of adrenal steroid patterns in man resulting from treatment with a chemical inhibitor of 11beta-hydroxylation. J Clin Endocrinol Metab 1958 18 906–912. (10.1210/jcem-18-8-906) 13563620

[2] Liddle GW, Estep HL, Kendall JW Jr, et al. Clinical application of a new test of pituitary reserve. J Clin Endocrinol Metab 1959 19 875–894. (10.1210/jcem-19-8-875) 14416789

[3] Jubiz W, Meikle AW, West CD, et al. Single-dose metyrapone test. Arch Intern Med 1970 125 472–474. (10.1001/archinte.1970.00310030082008) 4313728

[4] Spiger M, Jubiz W, Meikle AW, et al. Single-dose metyrapone test: review of a four-year experience. Arch Intern Med 1975 135 698–700. (10.1001/archinte.1975.00330050072012) 1052666

[5] Courtney CH, McAllister AS, McCance DR, et al. The insulin hypoglycaemia and overnight metyrapone tests in the assessment of the hypothalamic–pituitary–adrenal axis following pituitary surgery. Clin Endocrinol 2000 53 309–312. (10.1046/j.1365-2265.2000.01093.x) 10971447

[6] English K, Inder WJ, Weedon Z, et al. Prospective evaluation of a week one overnight metyrapone test with subsequent dynamic assessments of hypothalamic–pituitary–adrenal axis function after pituitary surgery. Clin Endocrinol 2017 87 35–43. (10.1111/cen.13334) 28329436

[7] Schöneshöfer M, Schefzig B & Arabin S. Short-term kinetics of serum adrenal steroids and plasma ACTH after a single dose of metyrapone in man. J Endocrinol Investig 1980 3 229–236. (10.1007/bf03348268) 6253555

[8] Laurberg P, Møller J, Husager L, et al. Evaluation of pituitary-adrenal function after pituitary surgery. Scand J Clin Lab Investig 1985 45 303–308. (10.3109/00365518509161011) 2990019

[9] Auzéby A, Bogdan A & Touitou Y. An alternate pathway to androstenedione synthesis by human adrenals: evidence of a balance in 11 beta-hydroxylase and 17,20-lyase activities leading to androstenedione. J Clin Endocrinol Metab 1995 80 1706–1711. (10.1210/jcem.80.5.7745023) 7745023

[10] Staub JJ, Noelpp B, Girard J, et al. The short metyrapone test: comparison of the plasma ACTH response to metyrapone and insulin-induced hypoglycaemia. Clin Endocrinol 1979 10 595–601. (10.1111/j.1365-2265.1979.tb02119.x) 225068

[11] Steiner H, Bähr V, Exner P, et al. Pituitary function tests: comparison of ACTH and 11-deoxy-cortisol responses in the metyrapone test and with the insulin hypoglycemia test. Exp Clin Endocrinol Diabetes 1994 102 33–38. (10.1055/s-0029-1211262) 8005206

[12] Kanaan E, Haase M, Vonend O, et al. Aldosterone-mediated sodium retention is reflected by the serum sodium to urinary sodium to (serum Potassium)2 to urinary potassium (SUSPPUP) index. Diagnostics 2020 10 545. (10.3390/diagnostics10080545) 32751768 PMC7460433

[13] Winzinger EP, Jandikova H, Haase M, et al. DHEAS and differential blood counts as indirect signs of glucocorticoid excess in adrenal non-producing adenomas. Horm Metab Res 2021 53 512–519. (10.1055/a-1539-6442) 34384108

[14] Fleseriu M, Hashim IA, Karavitaki N, et al. Hormonal replacement in hypopituitarism in adults: an Endocrine Society Clinical Practice Guideline. J Clin Endocrinol Metab 2016 101 3888–3921. (10.1210/jc.2016-2118) 27736313

[15] Campana C, Patelli I, Arecco A, et al. Hypopituitarism in patients with prolactinomas: a narrative review. Best Pract Res Clin Endocrinol Metabol 2026 40 102082. (10.1016/j.beem.2026.102082) 41638953

[16] Urhan E & Karaca Z. Diagnosis of hypoprolactinemia. Rev Endocr Metab Disord 2024 25 985–993. (10.1007/s11154-024-09896-8) 39037546 PMC11624249

[17] Toledano Y, Lubetsky A & Shimon I. Acquired prolactin deficiency in patients with disorders of the hypothalamic–pituitary axis. J Endocrinol Investig 2007 30 268–273. (10.1007/bf03346292) 17556861

[18] Molitch ME, Clemmons DR, Malozowski S, et al. Evaluation and treatment of adult growth hormone deficiency: an Endocrine Society Clinical Practice Guideline. J Clin Endocrinol Metab 2011 96 1587–1609. (10.1210/jc.2011-0179) 21602453

[19] DeLong ER, DeLong DM & Clarke-Pearson DL. Clarke-Pearson DL comparing the areas under two or more correlated receiver operating characteristic curves: a nonparametric approach. Biometrics 1988 44 837–845. (10.2307/2531595) 3203132

[20] Arlt W & Allolio B. Adrenal insufficiency. Lancet 2003 361 1881–1893. (10.1016/S0140-6736(03)13492-7) 12788587

[21] Nilsson B, Gustavsson-Kadaka E, Bengtsson BA, et al. Pituitary adenomas in Sweden between 1958 and 1991: incidence, survival, and mortality. J Clin Endocrinol Metab 2000 85 1420–1425. (10.1210/jcem.85.4.6498) 10770176

[22] Tomlinson JW, Holden N, Hills RK, et al. Association between premature mortality and hypopituitarism. West Midlands Prospective Hypopituitary Study Group. Lancet 2001 357 425–431. (10.1016/s0140-6736(00)04006-x) 11273062

[23] Wass JA & Arlt W. How to avoid precipitating an acute adrenal crisis. BMJ 2012 345 e6333. (10.1136/bmj.e6333) 23048013

[24] Charmandari E, Nicolaides NC & Chrousos GP. Adrenal insufficiency. Lancet 2014 383 2152–2167. (10.1016/s0140-6736(13)61684-0) 24503135

[25] Seoudy AK, Schlicht K, Kulle A, et al. Prospective analysis of the metyrapone short test using targeted and untargeted metabolomics. Neuroendocrinology 2023 113 770–784. (10.1159/000529146) 36646062

[26] Giordano R, Picu A, Bonelli L, et al. Hypothalamus–pituitary–adrenal axis evaluation in patients with hypothalamo-pituitary disorders: comparison of different provocative tests. Clin Endocrinol 2008 68 935–941. (10.1111/j.1365-2265.2007.03141.x) 18031311

[27] Noe S, von Werder A, Iakoubov R, et al. Dynamics of adrenocorticotropin after application of metyrapone. Exp Clin Endocrinol Diabetes 2017 125 53–56. (10.1055/s-0042-117832) 27750352

[28] Papierska L, Rabijewski M, Migda B, et al. Evaluation of plasma ACTH in the metyrapone test is insufficient for the diagnosis of secondary adrenal insufficiency. Front Endocrinol 2022 13 1004129. (10.3389/fendo.2022.1004129) PMC9684459 36440206

[29] Greene LW, Geer EB, Page-Wilson G, et al. Assay-specific spurious ACTH results lead to misdiagnosis, unnecessary testing, and surgical Misadventure-A case series. J Endocr Soc 2019 3 763–772. (10.1210/js.2019-00027) 30963134 PMC6446888

[30] Javorsky BR, Raff H, Carroll TB, et al. New cutoffs for the biochemical diagnosis of adrenal insufficiency after ACTH stimulation using specific cortisol assays. J Endocr Soc 2021 5 bvab022. (10.1210/jendso/bvab022) 33768189 PMC7975762

[31] Kazlauskaite R, Evans AT, Villabona CV, et al. Corticotropin tests for hypothalamic–pituitary–adrenal insufficiency: a metaanalysis. J Clin Endocrinol Metab 2008 93 4245–4253. (10.1210/jc.2008-0710) 18697868

[32] Berneis K, Staub JJ, Gessler A, et al. Combined stimulation of adrenocorticotropin and compound-S by single dose metyrapone test as an outpatient procedure to assess hypothalamic–pituitary–adrenal function. J Clin Endocrinol Metab 2002 87 5470–5475. (10.1210/jc.2001-011959) 12466339

[33] Pasquali R, Ambrosi B, Armanini D, et al. Cortisol and ACTH response to oral dexamethasone in obesity and effects of sex, body fat distribution, and dexamethasone concentrations: a dose-response study. J Clin Endocrinol Metab 2002 87 166–175. (10.1210/jcem.87.1.8158) 11788642

[34] Ness-Abramof R, Nabriski D, Apovian CM, et al. Overnight dexamethasone suppression test: a reliable screen for Cushing’s syndrome in the obese. Obes Res 2002 12 1217–1221. (10.1038/oby.2002.166) 12490665

[35] Nieman LK. Recent updates on the diagnosis and management of Cushing’s syndrome. Endocrinol Metab 2018 33 139–146. (10.3803/enm.2018.33.2.139) PMC6021313 29947171

[36] Ceccato F, Artusi C, Barbot M, et al. Dexamethasone measurement during low-dose suppression test for suspected hypercortisolism: threshold development with and validation. J Endocrinol Investig 2020 43 1105–1113. (10.1007/s40618-020-01197-6) 32060745

[37] Schmidt IL, Lahner H, Mann K, et al. Diagnosis of adrenal insufficiency: evaluation of the corticotropin-releasing hormone test and Basal serum cortisol in comparison to the insulin tolerance test in patients with hypothalamic–pituitary–adrenal disease. J Clin Endocrinol Metab 2003 88 4193–4198. (10.1210/jc.2002-021897) 12970286

[38] Erturk E, Jaffe CA & Barkan AL. Evaluation of the integrity of the hypothalamic–pituitary–adrenal axis by insulin hypoglycemia test. J Clin Endocrinol Metab 1998 83 2350–2354. (10.1210/jcem.83.7.4980) 9661607

[39] Benson S, Neumann P, Unger N, et al. Effects of standard glucocorticoid replacement therapies on subjective well being: a randomized, double blind, crossover study in patients with secondary adrenal insufficiency. Eur J Endocrinol 2012 167 679–685. (10.1530/eje-12-0351) 22930487

[40] Carosi G, Malchiodi E, Ferrante E, et al. Hypothalamic–pituitary axis in non-functioning pituitary adenomas: focus on the prevalence of isolated central hypoadrenalism. Neuroendocrinology 2015 102 267–273. (10.1159/000430815) 25924873

[41] Kienitz T, Bechmann N, Deutschbein T, et al. Adrenal crisis – definition, prevention and treatment: results from a Delphi survey. Horm Metab Res 2024 56 10–15. (10.1055/a-2130-1938) 37562416

